# Fracture pattern characteristics and associated injuries of high-energy, large fragment, partial articular radial head fractures: a preliminary imaging analysis

**DOI:** 10.1007/s10195-014-0331-x

**Published:** 2014-12-27

**Authors:** John T. Capo, Ben Shamian, Ramces Francisco, Virak Tan, Jared S. Preston, Linda Uko, Richard S. Yoon, Frank A. Liporace

**Affiliations:** 1 Division of Hand Surgery, Department of Orthopaedic Surgery, NYU Hospital for Joint Diseases, New York, NY 10009 USA; 2 Division of Hand and Microvascular Surgery, Department of Orthopaedic Surgery, Rutgers New Jersey Medical School, Newark, NJ USA; 3 Division of Orthopaedic Trauma, Department of Orthopaedic Surgery, NYU Hospital for Joint Diseases, 301 E 17th Street Suite 1402, New York, NY 10003 USA

**Keywords:** Radial head fracture, Coronoid fracture, Radial head fragment, Elbow dislocation

## Abstract

**Background:**

High-energy radial head injuries often present with a large partial articular displaced fragment with any number of surrounding injuries. The objective of the study was to determine the characteristics of large fragment, partial articular radial head fractures and determine any significant correlation with specific injury patterns.

**Materials and methods:**

Patients sustaining a radial head fracture from 2002−2010 were screened for participation. Twenty-five patients with documented partial articular radial head fractures were identified and completed the study. Our main outcome measurement was computed tomography (CT)-based analysis of the radial head fracture. The location of the radial head fracture fragment was evaluated from the axial CT scan in relation to the radial tuberosity used as a reference point. The fragment was characterized by location as anteromedial (AM), anterolateral (AL), posteromedial (PM) or posterolateral (PL) with the tuberosity referenced as straight posterior. All measurements were performed by a blinded, third party hand and upper extremity fellowship trained orthopedic surgeon. Fracture pattern, location, and size were then correlated with possible associated injuries obtained from prospective clinical data.

**Results:**

The radial head fracture fragments were most commonly within the AL quadrant (16/25; 64 %). Seven fracture fragments were in the AM quadrant and two in the PM quadrant. The fragment size averaged 42.5 % of the articular surface and spanned an average angle of 134.4^°^. Significant differences were noted between AM (49.5 %) and AL (40.3 %) fracture fragment size with the AM fragments being larger. Seventeen cases had associated coronoid fractures. Of the total 25 cases, 13 had fracture dislocations while 12 remained reduced following the injury. The rate of dislocation was highest in radial head fractures that involved the AM quadrant (6/7; 85.7 %) compared to the AL quadrant (7/16; 43.7 %). No dislocations were observed with PM fragments. Ten of the 13 (78 %) fracture dislocations had associated lateral collateral ligament (LCL)/medial collateral ligament tear. The most common associated injuries were coronoid fractures (68 %), dislocations (52 %), and LCL tears (44 %).

**Conclusion:**

The most common location for partial articular radial head fractures is the AL quadrant. The rate of elbow dislocation was highest in fractures involving the AM quadrant. Cases with large fragment, partial articular radial head fractures should undergo a CT scan; if associated with >30 % or >120^°^ fracture arc, then the patient should be assessed closely for obvious or occult instability. These are key associations that hopefully greatly aid in the consultation and preoperative planning settings.

**Level of evidence:**

Diagnostic III.

## Introduction

Radial head fractures commonly occur in the adult population and mostly affect active individuals between 20 and 60 years of age and account for one-third of all fractures of the elbow [1]. In the elbow, the radial head plays an important role as a secondary constraint during valgus stress in addition to its nearly circumferential articulation with the capitellum and the proximal radioulnar joint (PRUJ) [2, 3]. Thus, fractures of the radial head are at risk for elbow stiffness, decreased range of motion, decreased quality of life, and instability, either with or without surgery. While most minimally displaced fracture patterns are treated non-operatively and often do well, more complex fracture patterns, either with or without surgery, have produced mixed results, fueling continued controversy in this academic arena [4–6].

Proper function of the elbow joint is significantly dependent on the level of stability available along the full arc of motion. Identification of possible instability is of paramount importance in regard to operative decision making and pre-operative planning especially in regard to injuries sustained from more high-energy mechanisms. While previous studies have shown a correlation between instability of the elbow and the amount of displacement and the size of the radial head fracture, there are few reports on associated injuries, especially in regard to specific fracture location within the radial head [3, 7, 8]. Specifically, correlation between the position of the fracture fragment in the radial head and concomitant associated injuries of the elbow have not been investigated. Any associations found would prove valuable in the initial consultation and pre-operative setting in order to expect and rule out commonly associated ligamentous or soft tissue injuries that may or may not be the cause of elbow instability, especially in cases involving high-energy mechanisms. The purpose of this study was to evaluate high-energy, partial articular radial head fractures and identify any associated injuries of the elbow in reference to fracture location in the radial head. The objectives of this study were to provide radiographic pilot data for potential future correlation to operative and clinical outcomes in a larger, multi-center study setting.

## Materials and methods

Between 2002 and 2010, 57 patients with documented radial head fractures seen at a level 1 trauma center were identified from the database of two attending orthopedic surgeons. Inclusion criteria for the study included acute injury, large fragment partial articular (AO/OTA 21B2) radial head fractures, high-energy mechanism (pedestrian struck, motor vehicle accident (MVA), fall from above a standing height, etc.), skeletally mature individuals, and complete radiographic work-up including three adequate views of the elbow and computed tomography (CT) of the affected elbow. For those cases that were deemed operative, criteria included limited range of movement (ROM) secondary to block of motion or gross instability, gross instability at any point during the arc of motion, high degree of comminution, and/or >50 % radial head involvement [9, 10]. Exclusion criteria included those who sustained injuries from a gunshot wound, a low-energy fall, those without cortical contact with the radial neck/shaft, and those with incomplete clinical or radiographic medical records. Blinded, third party personnel collected demographic and operative data which was stored in a password-protected electronic database. The average age of the 25 patients was 43 years (range 19–72), with the injury involving the right side in 11 cases and the left side in 14 cases. Twenty-four cases were secondary to fall and one was due to an MVA (Table 1).

**Table 1:** Demographic data of study cohort

Case	Age/sex	Mechanism of injury	Side affected	Associated injuries with radial head fracture	Treatment
1	37/M	Fall	Left	Radial shaft fx, elbow dislocation	ORIF (plate and screws), LCL/MCL repair
2	54/F	Fall	Right	Coronoid fx, elbow dislocation	Radial head arthroplasty, ORIF coronoid, MCL/LCL repair
3	33/M	Fall	Left	Monteggia fx	Open reduction, ORIF multiple screws
4	59/F	Fall	Right	Elbow dislocation	Hinged external fixator
5	37/M	Fall	Right	Coronoid fx	ORIF radial head with multiple screws
6	47/F	Fall	Right	Coronoid fx, LCL tear	Radial head arthroplasty, LCL repair
7	19/M	Fall	Left	Radial head fx, coronoid fx, elbow dislocation	ORIF radial head/neck fx with plate and screws, ORIF coronoid
8	46/F	Fall	Right	Elbow dislocation, distal radius fx	ORIF radial head w/plate and screws, application of external fixator, LCL repair, percutaneous pinning of distal radius fx
9	39/F	Fall	Right	Elbow dislocation	Elbow arthroscopy, LCL repair
10	28/M	Fall	Left	Coronoid fx	Manipulation under anesthesia, closed treatment
11	41/F	Fall	Left	None	Sling, early ROM
12	59/F	Fall	Left	LCL tear	ORIF radial head with multiple screws, LCL repair
13	60/F	Fall	Left	Capitellar fx, coronoid fx, chondral injury, elbow dislocation	Total elbow arthroplasty, ulnar nerve transposition
14	41/M	MVA	Right	Ulna fx, MC fx dislocation, elbow dislocation	ORIF radial head with multiple screws, LCL repair
15	53/M	Fall	Left	Elbow dislocation, scaphoid fx, distal radius fx	Radial head arthroplasty, ORIF coronoid fx, LCL repair, application of external fixator
16	39/M	Fall	Right	None	Cast
17	22/M	Fall	Left	Coronoid fx, LCL tear	Radial head arthroplasty, ORIF coronoid, LCL repair
18	33/M	Fall	Left	Capitellar fx	Radial head arthroplasty
19	58/M	Fall	Right	Elbow dislocation, coronoid fx, LCL/MCL tear	Radial head arthroplasty, ORIF coronoid, LCL/MCL repair, hinged external fixator
20	52/F	Fall	Right	LCL tear, coronoid fx	Radial head arthroplasty
21	20/M	Fall	Left	Elbow dislocation, LCL tear, coronoid fx	ORIF radial head, LCL repair
22	72/F	Fall	Left	Coronoid fx	Radial head arthroplasty
23	33/M	Fall	Left	Capitellar fx	Radial head arthroplasty
24	49/F	Fall	Right	Elbow dislocation, LCL tear	ORIF with external fixator, LCL repair
25	44/M	Fall	Left	Olecranon fx, coronoid fx	ORIF of radial head, coronoid, and olecranon

*M* male, *F* female, *MVA* motor vehicle accident, *fall* high-energy fall, *fx* fracture, *LCL* lateral collateral ligament, *MCL* medial collateral ligament, *MC* metacarpal, *ORIF* open reduction and internal fixation, *ROM* range of motion

### Imaging analysis

**Fig. 1:**
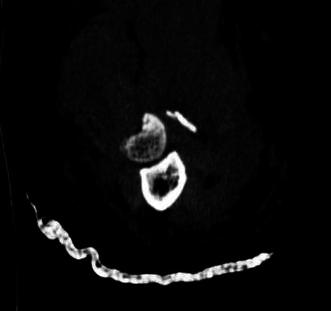
CT scan of unicondylar radial head fracture. Example of an axial cut that best revealed the largest diameter of the intact portion of the radial head and associated fracture defect used to quantify the fracture

**Fig. 2:**
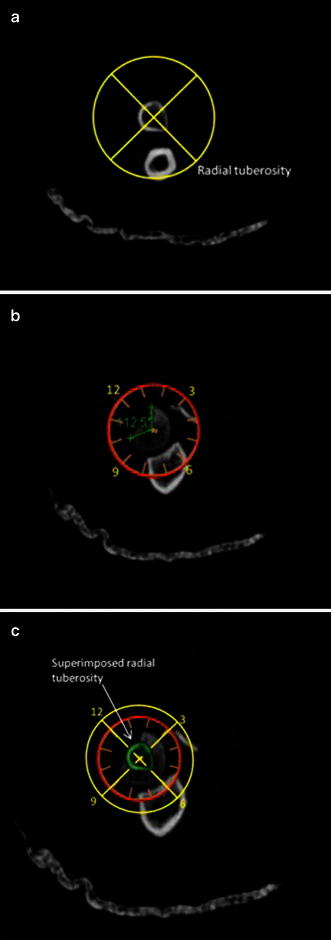
**a** Axial CT scan best representing the radial tuberosity. **b** Axial CT scan of radial head with superimposed clock face and measured angles of missing radial head fracture. **c** Axial CT scan of radial head and superimposed radial tuberosity

**Fig. 3:**
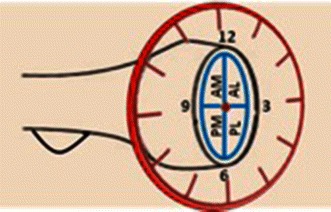
Line drawing of a right radial head and tuberosity with overlaid clock face demonstrating the various locations of radial head fractures. Note that radial tuberosity is assigned as 6 o’clock. *AM* anteromedial; *AL* anterolateral; *PM* posteromedial; *PL* posterolateral

All imaging modalities were reviewed by a single fellowship-trained hand and upper extremity, attending physician (JTC). All radiographic data were de-identified prior to analysis, in an attempt to minimize bias. CT scans of the elbow with 1-mm slice thickness were evaluated for the position and size of the fracture fragment in the radial head. Of note, if involved with concurrent elbow dislocation, CT scans were obtained post-reduction and splinting. The axial cut that best revealed the largest diameter of the intact portion of the radial head and associated fracture defect was used to quantify the fracture (Fig. 1). The axial cut that best demonstrated the radial tuberosity was then used as a reference marker to determine the angular location of the fracture (Fig. 2a). The radial tuberosity, designated as the 6 o’clock position, was utilized as a bony marker that was deemed as the most consistent landmark that is least likely to change in the setting of high-energy trauma, which may obscure and change soft tissue position. These selected images were then placed in a Microsoft Powerpoint (Microsoft Corp., Redmond, WA, USA) file. A computer-generated calibrated dial was then created and used as a measurement guide (Fig. 2b). Using the radial tuberosity as a reference point, the calibrated dial was then superimposed on the CT scan cut that best depicted the radial head fracture (Fig. 2c). The radial head fractures were then quantified as an angular amount and as a range on the face of a clock. Fracture fragment anatomy was termed in a specific quadrant based on location on the clock face—fractures between the 12 o’clock and 3 o’clock position were referred to as anterolateral (AL) fragments, between 3 o’clock and 6 o’clock position as posterolateral (PL) fragments, between 6 o’clock and 9 o’clock as posteromedial (PM) fragment, and between 9 o’clock and 12 o’clock as anteromedial (AM) fragments (Fig. 3). Fracture fragments spanning two adjacent quadrants were reported based on the larger fracture fragment component, which was quantified via measurement and percentage comparison. The exact angle was measured using a picture archiving and communication systems (PACS, General Electric Company, Fairfield, CT, USA) angle measuring tool. The percent amount of articular surface involvement in the radial head, and the presence of associated elbow fractures, elbow dislocation/subluxation and other associated injuries were also noted and recorded. The percent amount of articular surface involved was estimated using computed measuring area software (SkyScan High-Resolution MicroCT System, Kontich, Belgium).

Statistical analysis was performed using Student’s *t* test, and Fisher’s exact test for categorical data. All statistics were analyzed via SPSS 18.0 (IBM Inc., Armonk, NY, USA); a *p*-value of ≤0.05 was considered significant.

## Results

Examination of the CT scans revealed fracture fragments in the AL (16), AM (7), and PM (2) quadrants. The average amount of radial head surface fractured was 42.5 % (10.8–58 %), spanning an average angle of 134.4^o^ (65.7°–175°) from the center of the radial head. Cases with fracture dislocation (13/25) had an average radial head surface area involvement of 42.7 % while those that remained reduced (12/25) following the injury had 42.3 % (*p* = 0.777). Mean fracture fragment size of AM fractures were significantly larger than the AL and PM fragments (49.5 % vs 40.3 %; *p* = 0.024).

The incidence of dislocation among these various fracture fragments revealed that 6 out of 7 AM fragments had a dislocation (85.7 %) while only 7 of the 16 AL fragments had an associated dislocation (43.7 %; *p* = 0.021). No dislocations were observed with PM fragments. Posterior dislocations were observed in 11 cases while two had PL dislocations. Of the 23 operative cases, 11 had lateral collateral ligament (LCL) tears while 3 had combined LCL/ medial collateral ligament (MCL) tears. Of the eleven LCL tears, 7 had radial head fractures in the AL quadrant and 4 in the AM quadrant. The distribution of LCL/MCL injuries were two radial head fractures in the AM and one in the AL quadrant.

Twenty-three out of 25 cases had associated injuries. Seventeen cases had a coronoid fracture, with 12 of these cases having a type I coronoid fracture and five with a type II fracture. The group with fracture dislocations had a similar incidence of coronoid fractures (9/13; 70 %) compared to the non-dislocation group [9/12 (75 %)]. Coronoid fractures when correlated with the position of the radial head fracture fragment revealed that the highest incidence of radial head fragments were AL (12/16; 75 %) compared to AM (4/7; 57 %) and PM (1/2; 50 %) fragments. With these small numbers, no statistical difference was noted between the incidence of coronoid fractures among the different fracture types—AM versus AL (*p* = 0.409), AL versus PM (*p* = 0.499), and AM versus PM (*p* = 0.858). No statistical difference was observed between the various fracture fragments and their association with LCL or MCL tears. Radial fracture fragment position, direction of dislocation and amount of surface area fractured are summarized in Table 2.

**Table 2:** Radial fracture fragment position, direction of dislocation and amount of surface area fractured

Case	Quadrant	Amount of articular surface fractured (%)	Arc of fracture fragment (°)	Direction of dislocation
1	AM	50.0	175	Posterior
2	AL	25.4	112.5	Posterior
3	AL	50.2	118.8	Posterior
4	AL	10.8	65.7	Posterior
5	AL	44.5	108.9	None
6	AL	40.0	131	None
7	AL	48.3	137.9	Posterolateral
8	AL	34.3	101.5	Posterolateral
9	AL	36.4	128.3	Posterior
10	AL	35.0	168.7	None
11	PM	31.7	151.5	None
12	AL	42.8	155.3	None
13	AM	42.1	146.2	Posterior
14	AM	40.7	141.2	Posterior
15	AM	55.5	126.3	Posterior
16	AL	33.3	114	None
17	PM	41.0	151.7	None
18	AM	50.2	162	None
19	AM	50.3	145	Posterior
20	AL	52.1	137.3	None
21	AM	58.1	157.6	Posterior
22	AL	50.2	167.3	None
23	AL	53.2	121.6	None
24	AL	53.2	111.2	Posterior
25	AL	35.2	124.7	None
Averages		42.5 %	134.4	

*AM* anteromedial, *AL* anterolateral, *PM* posteromedial, *PL* posterolateral

## Discussion

Radial head fractures are common and may be associated with other injuries of clinical importance, especially when secondary to high-energy traumatic mechanisms. As well as the specific fracture pattern, associated humeroulnar dislocation, ligament disruption and other associated elbow fractures must be considered when evaluating fractures of the radial head. The extent of bony involvement, associated fractures and soft tissue injury helps to determine appropriate management of these complex injuries [11–13].

Kaas et al. evaluated 44 patients with 46 radial head fractures for associated soft-tissue injuries. The radial head injuries included 17 Mason type I fractures, 23 Mason type II fractures, and 6 Mason type III fractures. Using only magnetic resonance imaging (MRI), associated injuries were documented in 35 elbows—28 elbows had LCL lesions, 18 had capitellar injuries, 1 had a coronoid fracture, and 1 had an MCL injury [14]. In our series, documented injuries associated with radial head fractures were also high, exhibiting a high rate of both bony (coronoid) and soft tissue (MCL/LCL) injuries.

van Riet et al. [6] also evaluated the frequency of associated injuries in 333 radial head fractures. Two hundred and twenty-three (67 %) patients had Mason type I fractures, 46 had Mason type II (14 %) fractures, and 64 had Mason type III (19 %) fractures. One hundred and eighteen of 333 patients (39 %) had associated fractures or soft-tissue injury. Fifty-three (16 %) patients had coronoid fractures, and 45 patients (14 %) had elbow dislocations. Thirty-five LCL injuries (11 %), 5 MCL injuries (2 %), and 20 injuries involving both the LCL and MCL (6 %) were also reported. In the study by van Riet et al., the likelihood of associated elbow injuries increased (*p* < 0.05) with fracture severity. Additional injuries occurred in 8 % of patients with Mason type I radial head fractures (17/223 patients), in 50 % with Mason type II fractures (23/46 patients), and in 75 % with Mason type III fractures (48/64 patients). Our series revealed similar findings with the highest associated injury being coronoid fractures (68 %).

In another study by Beingessner et al. [7] the effect of radial head fracture size on radiocapitellar stability was examined. In their study, fractures were simulated in six fresh-frozen cadaveric radiocapitellar joints by sequential removal of 20° wedges from the AL aspect of each radial head until 140° of the radial head was removed. Decreased shear load at the radial head during joint loading was used as an indicator of decreased stability at the radiocapitellar joint. There was no difference in the shear load between the intact specimen and that with a 20° wedge removed (*p* > 0.05). However, stability decreased with each increase in wedge size between 20° and 120° (*p* < 0.05). Beyond 120° of wedge removal, which is one-third of the diameter of the radial head, the shear load was constantly low, indicating lower stability. They concluded that an inverse relationship exists between radiocapitellar joint stability and radial head fracture size [7]. In our series, regardless of fracture location, utilizing the aforementioned data provided by Beingessner et al., 18 fractures consisted of an arc >120°, of which 16 (89 %) were deemed unstable (i.e., concomitant dislocation, radiocapitallar instability, ligamentous injury, etc., (Tables 1, 2). While this correlation may be biased to the underpowered nature of our study, the trend is still worth noting that with a higher arc of fracture, a high incidence of concomitant instability is likely present.

In a study similar to ours, van Leeuwen investigated Mason type II fractures using 3-dimensional CT. Both studies showed fractures were most often found throughout the AL portion of the radial head. However, our study showed a wider distribution of fractures throughout the AM quadrant, whereas van Leeuwen conversely reported more patients with fractures through the AL portion. We found fractures to span a smaller arc, whereas van Leeuwen found the fractures to span 170^o^. van Leeuwen did not comment on injuries associated with Mason type II fractures or the percent involvement of the articular surface.

Radial head fractures can occur in isolation, or with associated ligament and bony injuries, which may further compromise elbow and forearm stability. Commonly, the amount of head involvement is used to determine the need for surgery in radial head fractures. Assessment of the size of the fragment must be combined with knowledge of associated injuries in planning appropriate treatment of these complex injuries. Associated injuries and their early knowledge may help guide treatment options that may vary from plate and screw fixation to prosthetic replacement [4, 15–18]. Furthermore, in the setting of the initial consultation, being aware of associated injuries that frequently occur has the potential to avoid missed injuries with a focus on looking for associated instability.

This study has several limitations. First and foremost, as a result of our strict inclusion and exclusion criteria, our overall number of study subjects is low and makes our study underpowered. This inhibited any type of regression analysis to notice specific trends (i.e., operative outcomes from associated fracture patterns and injuries). Furthermore, while the advantages of a single surgeon imaging analysis provides uniformity and removes some bias, lack of reliability calculations provides a scenario that may have provided lack of agreement should another observer have been possible. The aforementioned limitations along with the lack of impact on operative decision-making along with correlation with intraoperative findings also limited our study. However, the objectives of this study were to build upon the reliability findings determined by van Leeuwen et al. [19] and to provide initial preliminary data which we hope to utilize in a prospective, large-scale, multi-center study that will provide the ability to perform regression and obtain operative decision-making capacity and intraoperative correlation. Definitive assessment on a larger scale will provide important data, and this initial data provided here is the first step.

The findings in our study are consistent with the often-mentioned fact that the AL portion is the most common location of a high-energy, large fragment, partial articular radial head fracture. CT scans should be obtained and fractures associated with a >30 % or >120° fracture arc should be analyzed closely for occult or obvious instability. The most common associated injuries in our study include coronoid fractures, elbow dislocation and LCL tears. The size of an AM fragment was on average larger than an AL fragment. In addition, AM fractures had a higher association with elbow dislocation than AL fractures of the radial head. A better understanding of the characteristics of these unicondylar radial head fractures may assist in improved treatment of these injuries. Finally, knowledge of associated injuries will be valuable in the consultation setting, with a focus on not missing these crucially concomitant injuries of the elbow. Our objective is to utilize this preliminary data as the foundation for a larger, multi-center prospective study with intraoperative correlation to further assess and definitively determine associated fractures and instability patterns.
